# Selected somatic parameters and body composition as predictors of cardiorespiratory fitness among Polish adolescents aged 11–14

**DOI:** 10.1038/s41598-024-75821-3

**Published:** 2024-10-25

**Authors:** Karolina Marks, Dorota Kopeć, Justyna Lenik, Paweł Lenik, Bartosz Dziadek

**Affiliations:** Institute of Physical Culture Sciences, Medical College of Rzeszów University, Rzeszów University, 35-959 Rzeszów, Poland

**Keywords:** 20 m shuttle run test, Primary school, Regression, Paediatrics, Population screening

## Abstract

The aim of the study was to verify whether selected somatic parameters and components of body composition were significant predictors of cardiorespiratory fitness (CRF) among a potentially healthy Polish population of adolescents aged 11–14 years. The cross-sectional study was conducted on a group of 375 subjects (164 girls, and 211 boys). A 20 m shuttle run test (20 m SRT) was used to assess CRF. The total number of rounds was taken into account. Basic somatic parameters were measured: body mass (BM), body height (BH), waist circumference (WC), hip circumference (HC), body mass index (BMI), waist-to-hip ratio (WHR) and waist-to-height ratio (WHtR), and body composition components: body fat percentage (FM%), fat mass (FM kg), total body water (TBW), fat-free mass (FFM). Statistical analyses included basic statistical measures (mean and standard deviation) and Spearman rank correlation coefficient. Multiple linear regression analysis was performed to detect significant predictors of CRF. In each proposed model, the dependent variable was the number of rounds, and the independent variables were selected somatic parameters and components of body composition. More than half (65%) of the subjects had an average or lower level of CRF, and 35% of the population presented a good or above good level of CRF. The study showed a statistically significant negative correlation between BM, FM%, FM kg, HC, WC, BMI, WHR, WHtR and the number of laps in the total sample. The strongest correlation in the group of girls was noted for age (r = 0.34) and in the group of boys for FM% (r = $-0.52$). Each regression model presented proved to be statistically significant, and the significant predictors of CRF in the group of girls were age ($R^2$ = 16%) and FM% ($R^2$ = 6%). In the group of boys, the significant predictors of CRF were WHtR ($R^2$ = 8%) and age ($R^2$ = 2%). Estimating body fat distribution is useful in assessing cardiorespiratory fitness, and this in turn indicates its usefulness in preventive screening of school-aged adolescents.

## Introduction

Cardiorespiratory fitness (CRF), also referred to as aerobic capacity, is a major indicator of cardiovascular health. High levels of CRF in childhood and adolescence translate positively into cardiovascular function during this period of life as well as in later life^1–3^. Children and adolescents with low CRF have a higher risk of cardiovascular disease and myocardial infarction in adulthood^2,4^. A decline in aerobic capacity from childhood to adolescence is also associated with an increased risk of overweight and metabolic syndrome in adults^5–7^. In addition to being a predictor of cardiovascular health among adolescents, CRF is also associated with academic achievement^8^ or mental health^9^.

The main indicator of CRF is the body’s maximum oxygen uptake capacity ($\hbox {VO}_{2}$max). $\hbox {VO}_{2}$max directly reflects the overall capacity of the cardiovascular and respiratory systems, as well as the ability to perform prolonged exercise^10^. Methods of measuring $\hbox {VO}_{2}$max can be divided into direct (laboratory methods) and indirect (field-based tests). The use of direct methods in schools or population studies is expensive and requires specialized equipment and properly trained personnel. The choice of indirect tests saves money and time, as they can be used to study a large group of individuals^11,12^.

There are a number of scientific publications in which the authors examine factors affecting CRF. According to Lakoski et al.^13^, age, sex, body mass index (BMI) and physical activity are the most important factors associated with CRF. Other authors have indicated that BMI is significantly and negatively correlated with the level of $\hbox {VO}_{2}$max^14,15^. In a study conducted on a population of Caucasian men, where the main objective was to evaluate the relationship between waist circumference (WC) and body fat and CRF, it was noted that men with high CRF had significantly lower WC parameters and body fat compared to a group of subjects with low CRF^16^. After studying a group of firefighters, Barry et al. concluded that physical activity and WC are significant predictors of CRF^17^. Analysis of covariance by Agostinis-Sobrinho et al. showed a significant association between changes in CRF and systolic blood pressure and pulse pressure product after adjusting for age, sex, body height, puberty, socioeconomic status and WC, among adolescents aged 12–18 years old^18^. The regression model developed by Zadarko-Domaradzka et al. showed that WC, in addition to FM% and WHtR, is a useful measure of CRF, stronger than BMI or WHR^19^. On the other hand, Sarpong et al. proved that there is a significant positive relationship between WHR and CRF^20^. We also find information that CRF is inversely correlated with all measures of body composition (weight, BMI, FM%, WC, HC and WHR) after adjusting for age and sex, and additionally, WC is most strongly associated with CRF^21^. Other studies indicate that skeletal muscle mass is more strongly correlated with CRF among adolescents than total body weight and might be a better scaling variable for estimating expected peak $\hbox {VO}_{2}$^22^. Similar results were obtained in a study conducted among obese Chinese population. Skeletal muscle mass was found to be significantly correlated with $\hbox {VO}_{2}$max after taking into account sex, age, BMI, WHR and FM%^23^. It turns out that WHR is a much more accurate measure of obesity than BMI^24^, which is particularly important in predicting cardiovascular disease (CVD), a major cause of mortality in developed countries^1,25^. Many studies confirm that body composition components influence on human functional capabilities. According to Saha, there is a correlation between body composition and $\hbox {VO}_{2}$max^26^. Bresdenyuk, who conducted research on a group of students aged 17–21, noticed that people with “low” and “normal” body fat content had “excellent” levels of aerobic capacity according to the Ya.P. Pyarnat’s criteria^27^.

CRF is considered one of the predictive risk factors for CVD in children and adolescents. Even though studies show that CVD most commonly affects people over 50 years of age^28^, there is emerging evidence that CVD symptoms originate in childhood and adolescence^29^. Therefore, early detection of CVD risk factors contributes to effective prevention programs, counselling and public health policies. The aim of this study was to examine whether selected somatic parameters and body composition components were significant predictors of cardiorespiratory endurance capacity among a potentially healthy population of adolescents aged 11–14 years. We had the following hypotheses: somatic parameters and body composition may probably affect the level of cardiorespiratory fitness due to differences in the proportions of individual body composition components^30,31^.

## Material and methods

### Sample

To determine the selected somatic parameters, body composition components and level of CRF of adolescents, a cross-sectional study was conducted. The study involved 375 Polish adolescents aged 11–14, including 164 girls and 211 boys. The group was homogeneous in terms of age. The average age for girls was $12.3 \pm 1.1$, while for boys it was $12.2 \pm 1.0$. The largest group was made up of 11-year-olds (31.7%), followed by 12-year-olds (about 27.4%), those aged 13 accounted for about 26.1% of the surveyed population, while the smallest group was 14-year-olds (14.6%).

All primary schools in the city of Rzeszów were invited to participate in the research. Ultimately, 4 primary schools agreed to participate in the project. Students who had no health contraindications to participate in school physical education classes took part in the study. Before starting the research, written informed consent was obtained from the parents of all the Children’s. Students whose parents did not consent to participate in the project and those who had consent but were absent on the days of the study were excluded from the study. Before taking part in the study, students and parents were informed about the purpose and course of the running test and the anthropometric measurements to be taken.

The research was conducted over a 2-month period (May/June 2023) in two stages. During the first stage (May 2023), body composition measurements were carried out, followed by anthropometric measurements. The measurements took place in a closed room where one student and the people taking the measurements were present at the same time (measurements were always carried out by the same two people). The measurements were carried out in conditions that ensured privacy and respect for the dignity of the examined children: they were measured individually, boys and girls separately. The results were recorded on measurement cards. The second stage of the research included measuring cardiorespiratory fitness (June 2024). The study was conducted in the morning, during physical education lessons, according to the schedule. All the participants were provided with identical conditions and the test was conducted in the school sports hall: 15 students participated in the running test at one time. The results were recorded on measurement cards.

The study was carried out by the Declaration of Helsinki and the terms of local legislation. The experimental protocol was evaluated and approved by the Ethics Committee of the University of Rzeszow/Poland (resolution 5/03/2020).

### Methods

#### Sample size calculation

Before starting research, the sample size was calculated. The sample size for multiple regression analysis was estimated using G*power software (v3.1.9.7). Assuming a medium effect size ($\hbox {F}^2$ = 0.15), significance level $\alpha$ = 0.05, and desired statistical power ($1-\beta$) of 0.90 for a maximum of 12 predictors in the regression model, the required sample size was N = 157.

#### Cardiorespiratory fitness

The 20 m shuttle run test (20 m SRT) was used to assess CRF in accordance with the ALPHA (Assessing Levels of Physical Activity)^32,33^ health-related fitness test battery protocol. Participants ran between 2 lines 20 m apart while keeping up with the sound signals emitted from the app. The starting speed of 8.5 km/h was increased by 0.5 km/h per min (1 min equals 1 stage). Participants were instructed to run in a straight line and move according to the sound signals. A participant ended the test when he or she failed to reach the end line on two consecutive occasions or when they stopped due to fatigue. For the test to run properly, the subjects ran with an instructor from the start of the test until the last participant completed the test. The 20 m shuttle run test is highly accurate^11^ and reliable^34^ in the evaluation of CRF in children and adolescents. Classification of CRF levels was made based on laps according to Tomkinson’s guidelines^35^. Seven levels of CRF were adopted, in which P stands for percentile: very poor P5, poor P20, fair P40, average P60, good P80, very good P95, excellent > P95.

#### Anthropometric measurements

Body height was measured to an accuracy of 1 mm, using a mobile stadiometer from SECA 2017 (Germany). The measurement took place without shoes, and the subject stood on the stadiometer platform with feet together and the heels in contact with the platform stop. After the head was properly positioned in the Frankfurt plane, the measuring arm of the stadiometer was moved in. The waist circumference (WC) measurement was performed midway between the bottom edge of the lower rib and the upper iliac crest. The hip circumference (HC) measurement was performed at the height of the crests of the greater femur (through the largest buttock prominence)^36^. Anthropometric measurements were taken with a fixed tension anthropometric tape measure. The results were recorded in cm with an accuracy of one decimal place. The following indices were calculated: body mass index (BMI kg/m$^2$), waist-to-hip ratio (WHR) and waist-to-height ratio (WHtR).

#### Body composition

Body composition was assessed using bioelectrical impedance analysis (Tanita DC430 MA S). Body mass (BM) and its components were measured: FM% body fat percentage, FM kg body fat in kilograms, FFM kg fat-free mass, and TBW kg total body water.

#### Statistical analysis

Basic statistical measures (e.g. mean and standard deviation) were used to characterise the study group in terms of anthropometry, body composition and other variables. Based on the analysis of the results of the Kolmogorov–Smirnov test to identify differences between groups of men and women, the following tests were used Mann–Whitney U test. The relationship between the number of laps in the 20 m shuttle run test and selected anthropometric parameters and body composition was determined using Spearman’s rank correlation.

Multiple linear regression was used to assess which of the studied parameters could be significant predictors of CRF. In each proposed model, the dependent variable was the number of laps in the 20m shuttle run test. Age, sex, the selected somatic parameters (BH, HC, WC), body composition components (BM, FFM, TBW, FM, FM%) and BMI, WHR and WHtR index were independent variables. The process of building the model began with examining the relationship between CRF and the predictors. Variables showing a statistically significant relationship with CRF were selected for the models for the boys and girls group. In the next step, the collinearity of independent variables was checked by eliminating from further analysis predictors that showed a strong correlation between themselves (r > 0, 80). Additionally, the variance inflation factor (VIF) and the multiple linear regression assumptions (linearity, independence, homoscedasticity and normality) for the proposed models were checked. To reduce heteroscedasticity, the log transformation of the dependent variable was performed^37–39^. The analysis included the following calculations: unstandardised beta ($\beta$), standard error (SE) and residual standard error (RSE). Adjusted R-squared (Adj. $\hbox {R}^2$) and statistical significance were calculated for the whole model and each variable included.

All statistical calculations were performed using R Statistical Software (v4.3.3)^40^ at a significance level of $\alpha$ = 0.05. The additional r2glmm R package (v0.1.2)^41^ was used to determine the partial $\hbox {R}^2$ of the variables building the model.

## Results

Table 1 shows the characteristics of the study group in terms of somatic indicators and CRF test results (number of laps). The average age in the group of boys and girls differed significantly. Significant differences (at the significance level $\alpha$ = 0.05) between girls and boys were also observed for BM, WC, WHR, FM%, TBW and FM kg parameters. The analysis also showed that boys achieved better results in the 20 m SRT compared to girls. This difference is statistically significant (p < 0.001).

**Table 1:** Characteristics of a group—comparison by sex.

Variable	Unit	Girls (N = 164)	Boys (N = 211)	p	Total (N = 375)
Age	[years]	12.3 ± 1.1	12.2 ± 1.0	< 0.001*	12.2 ± 1.1
BH	[cm]	154.4 ± 8.7	155.5 ± 10.9	0.387	155.0 ± 10.0
BM	[kg]	45.8 ± 10.6	48.4 ± 13.2	< 0.001*	47.3 ± 12.2
BMI	[$\hbox {kg/m}^2$]	19.0 ± 3.0	19.7 ± 3.8	0.872	19.4 ± 3.5
HC	[cm]	82.4 ± 8.2	81.7 ± 9.3	0.667	82.0 ± 8.8
WC	[cm]	61.4 ± 7.0	66.1 ± 9.7	< 0.001*	64.0 ± 8.9
WHR	–	0.75 ± 0.04	0.81 ± 0.05	< 0.001*	0.78 ± 0.08
WHtR	–	0.40 ± 0.04	0.42 ± 0.05	0.064	0.41 ± 0.05
FFM	[kg]	35.6 ± 5.5	40.7 ± 9.9	0.061	38.4 ± 8.6
TBW	[kg]	26.0 ± 4.0	29.6 ± 6.8	0.048*	28.0 ± 6.8
FM	[kg]	10.1 ± 5.5	8.1 ± 5.8	< 0.001*	9.0 ± 5.8
FM%	[%]	20.9 ± 7.6	15.4 ± 7.4	< 0.001*	17.8 ± 8.0
Number of laps	–	28.7 ± 11.8	38.5 ± 17.4	< 0.001*	34.2 ± 16.0

*BH* body height, *BM* body mass, *BMI* body mass index, *HC* hip circumference, *WC* waist circumference, *WHR* waist-to-hip ratio, *WHtR* waist-to-height ratio, *FFM* fat-free mass, *TBW* total body water, *FM* body fat in kilograms, *FM*% body fat percentage.

*Statistical significance $$\alpha$$ = 0.05

Analyzing the CRF level classifications in relation to the number of laps (Table 2) in the study population of adolescents, it is noted that among girls the leading level of CRF was the level of fair (about 35%), followed by average (about 21%) and good (about 18%). In the group of boys, the distribution of CRF levels is different, as about 21% of boys were characterized by CRF at the level of poor and average CRF. The most represented level of CRF in boys was very good with about 30%. Analyzing the entire study population, about 27% of the adolescents achieved a fair level in the CRF test, slightly more than 21% reached the average level, and about 18% were at the good level. Only about 3% of the study population presented the highest level of CRF.

**Table 2:** Classification of CRF in groups of boys and girls.

Classification of CRF	Sex		Total
	Girls	Boys	
Very poor (< P5)	0 (0.0)	3 (1.4)	3 (0.8)
Poor (P20)	18 (11.0)	45 (21.3)	63 (16.8)
Fair (P40)	57 (34.8)	44 (20.9)	101 (26.9)
Average (P60)	34 (20.7)	45 (22.7)	79 (21.1)
Good (P80)	30 (18.3)	38 (18.0)	68 (18.1)
Very good (P95)	19 (11.6)	28 (30.2)	47 (12.5)
Excellent (> P95)	6 (3.7)	6 (2.8)	12 (3.2)
Total	164 (43.7)	211 (56.3)	375 (100.0)

Analysis of correlation coefficients (Table 3) between CRF Level and body composition and somatic parameters in total sample showed statistically significant negative correlations between BM, BMI, HC, WC, WHR, WHtR, FM (% and kg) and CRF level (p < 0.05). The strongest significant correlation, at r = $-0.51$, was recorded between FM% and CRF level. Analyzing the results in the group of girls, it is noted that the strongest significant correlation was noted with age (r = 0.34), WHtR (r = $-0.26$) and FM% (r = $-0.27$). In the group of boys, the results are different: the strongest significant correlation was noted with FM% (r = $-0.52$), then with FM kg and WHtR (r = $-0.44$), BMI (r = $-0.34$) and WC and WHR (r = $-0.31$).

**Table 3:** The correlation coefficient for independent variables and the number of laps in the 20 m shuttle run test.

Independent variables	Girls	Boys	Total
Age [years]	0.34*	0.17*	0.19*
BH [cm]	0.14	0.10	0.12*
BM [kg]	$-0.04$	$-0.17$*	$-0.11$*
BMI [$\hbox {kg/m}^2$]	$-0.19$*	$-0.34$*	$-0.26$*
HC [cm]	$-0.05$	$-0.23$*	$-0.18$*
WC [cm]	$-0.14$	$-0.31$*	$-0.16$*
WHR	$-0.15$*	$-0.31$*	$-0.02$
WHtR	$-0.26$*	$-0.44$*	$-0.26$*
FFM [kg]	0.10	0.02	0.09
TBW [kg]	0.10	0.01	0.09
FM [kg]	$-0.19$*	$-0.44$*	$-0.41$*
FM [%]	$-0.27$*	$-0.52$*	$-0.51$*

*BH* body height, *BM* body mass, *BMI* body mass index, *HC* hip circumference, *WC* waist circumference, *WHR* waist-to-hip ratio, *WHtR* waist-to-height ratio, *FFM* fat-free mass, *TBW* total body water, *FM* body fat, *FM*% body fat percentage.

*Statistical significance $$\alpha =0.05$$.

**Fig. 1:**
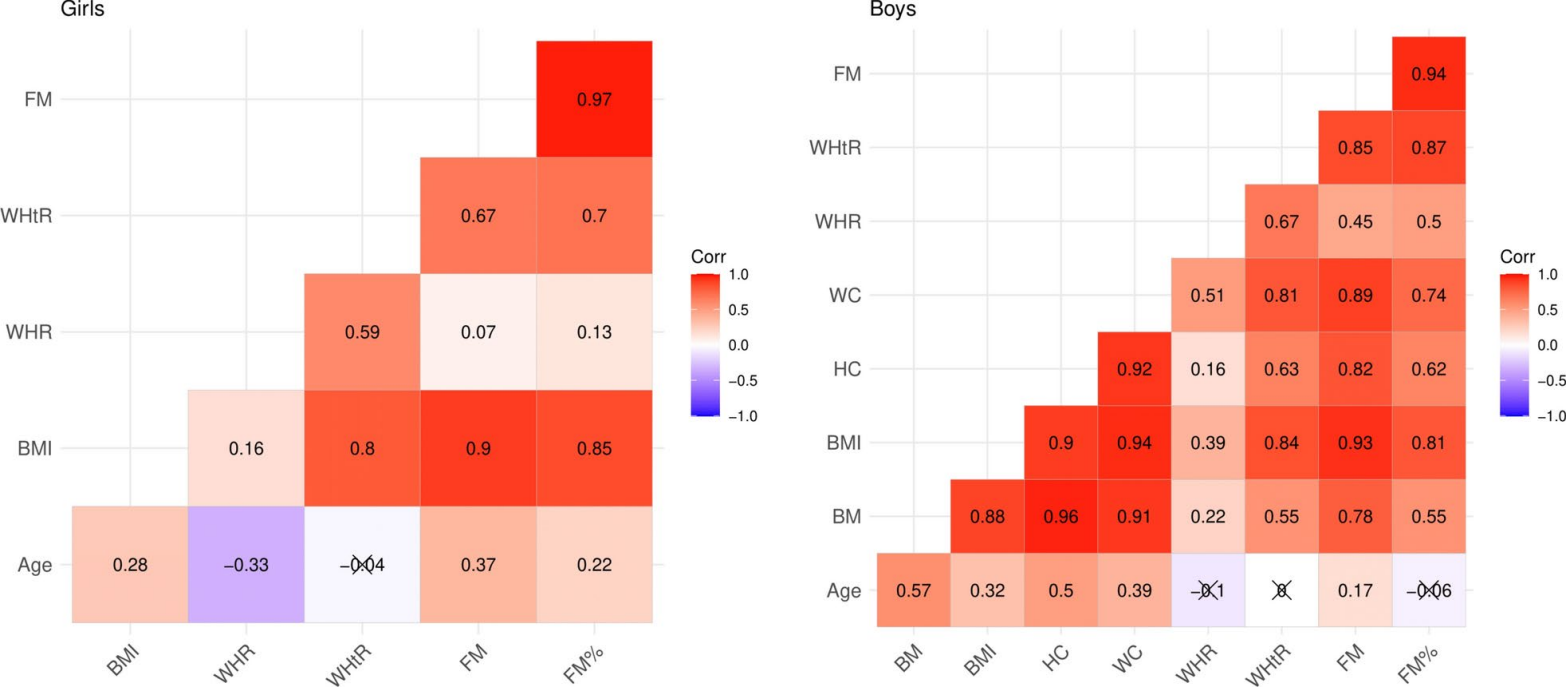
Correlation values between independent variables for the group of girls and boys.

Taking into account only statistically significantly correlated variables with the CRF level in further analysis, the collinearity of dependent variables was assessed (Fig. 1). The variables strongly correlated (r > = 0.80) with other predictors, i.e. BMI, FM in the girl’s group and BM, BMI, HC, WC, FM and FM% in the boy’s group, were not taken into account in the construction of the final version of the models.

Table 4 shows the results of the multiple regression models describing the relationship between CRF and selected independent variables in a group of girls. In the proposed models, the dependent variable was the number of laps, and the selected independent variables included: age, WHR, WHtR and FM%. The analysis showed that the presented model was statistically significant (p < 0.001) and the coefficient of determination was 22%. The somatic parameters and body composition components with statistical significance in this regression model were age (p < 0.001) and FM% (p < 0.001). The VIF (Variance Inflation Factor) values for predictors in this model were below 10, and the highest was 5.88 concerned with the waist-to-height ratio coefficient for girls.

**Table 4:** Multiple regression models describing the relationship between CRF and selected independent variables—Girls.

Independent variable	$\beta$	SE	p. $R^2$	VIF	p	RSE	Adj. $R^2$	F	p
Age	0.17	0.03	0.16	1.21	< 0.001*	0.36	0.22	12.34	< 0.001*
WHR	0.01	0.05	0.00	2.96	0.812				
WHtR	0.02	0.07	0.00	5.80	0.731				
FM%	$-0.17$	0.05	0.06	3.45	0.001*				

$\beta$ unstandardized beta, *SE* standard error, p. $R^2$ partial R-squared, *VIF* Variance Inflation Factor, *p* statistical probability, *RSE* Residual Standard Error, Adj. $R^2$ Adjusted R-squared, *WHR* waist-to-hip ratio, *WHtR* waist-to-height ratio, *FM%* body fat percentage.

*Statistical significance $$\alpha =0.05$$.

The somatic parameters and body composition components with the statistical significance in the multiple regression models in the group of boys (Table 5) for the number of laps was age (p = 0.043) and WHtR (p < 0.001). The predictive value of this model was similar to the group of girls ($R^2$ = 21%). The VIF analysis results (VIF < 5) don’t indicate a multicollinearity problem for this model.

**Table 5:** Multiple regression models describing the relationship between CRF and selected independent variables—Boys.

Independent variable	$\beta$	SE	p. $R^2$	VIF	p	RSE	Adj. $R^2$	F	p
Age	0.09	0.04	0.02	1.99	0.043*	0.45	0.21	15.14	< 0.001*
BM	0.03	0.06	0.00	3.38	0.603				
WHR	0.05	0.05	0.01	2.90	0.317				
WHtR	$-0.28$	0.07	0.08	4.81	< 0.001*				

$\beta$ unstandardized beta, *SE* standard error, p. $R^2$ partial R-squared, *VIF* Variance Inflation Factor, *p* statistical probability, *RSE* Residual Standard Error, Adj. $R^2$ Adjusted R-squared, *BM* body mass, *WHR* waist-to-hip ratio, *WHtR* waist-to-height ratio.

*Statistical significance $$\alpha =0.05$$.

## Discussion

The aim of the study was to verify whether selected somatic parameters such as BM, WHR, WHtR, BMI and body composition components (FFM kg, FAT% and kg, TBW kg) were significant predictors of cardiorespiratory fitness among a potentially healthy population of adolescents aged 11–14 years. Anthropometric measurements indicated that girls were characterized by higher body fat, while boys had higher fat-free mass and body water content. No statistically significant differences were found between the two sexes in terms of BMI, HC, FFM and WHtR. The study also showed significant differences between girls and boys in terms of CRF measurements. Boys obtained better results than girls. However, looking at the general population studied, the overall percentage of school-aged adolescents below good CRF levels (P60–P80) was as high as 65%.

Analyzing the general population, it is found that there is a statistically significant negative correlation between BM, BMI, HC, WC, WHR, WHtR, FM, FM% and CRF levels. The strongest negative correlation between body composition and CRF level, among the total study population, was noted for FM% (r = $-0.51$), FM kg (r = $-0.41$), WHtR (r = $-0.26$) and BMI (r = $-0.26$). Paying attention to the sex of the subjects, it is found that in both girls and boys, the CRF level correlates most strongly and negatively with FM% (respectively r = $-0.27$; r = $-0.52$). However, it should be noted, that the strongest correlation in the group of girls was noted with regard to age. The correlation in this case had a positive direction r = 0.34. Comparing the correlation between FM% and CRF level in this study to the studies conducted by other authors, similar relationships are observed. A similar correlation, in the boys’ group, was noted by Maciejczyk et al. (r = $-0.40$) and Amani et al. (r = $-0.40$)^42,43^. In the study by Ekelund et al. and Mota et al. the correlation between FM% and $\hbox {VO}_{2}$max also took similar values, i.e. r = $-0.48$ and r = $-0.49$ respectively^44,45^. Similar conclusions were reached by Cao et al. studying a population of Japanese adults. According to the authors, age, BMI, WC and FM% were significantly related to $\hbox {VO}_{2}$max (partial correlation coefficient r = $-0.54$, r = $-0.31$, r = $-0.55$, and r = $-0.64$, respectively)^46^. These results are partly confirmed by the research of Stojanovic^47^. Although the authors noted a significant correlation between FM% and $\hbox {VO}_{2}$max, only in the boys’ group did the correlation take a negative direction (r = $-0.53$). In the group of girls, they noted a statistically significant positive correlation (r = 0.45). Other studies indicate that there is no significant correlation between FM% and $\hbox {VO}_{2}$max^48,49^. In addition, the present study finds that FFM kg did not correlate significantly with the CRF level (r = $-0.08$). This phenomenon is observed regardless of the gender of the subjects. These results are not confirmed by the studies of other authors, who noted a significant correlation between FFM and CRF^47,50^.

When determining CRF predictors, many authors mainly took into account body composition components related to the content of adipose tissue in the body and created regression models based on these components^19,42,45,51^. Emadeldin et al. and Mondal et al. also presented similar regression models in their works^50,52^. The analysis showed that components such as age, WHR, WHtR and FM% (group of girls) and age, BM, WHR and WHtR (group of boys), explained the number of laps by, respectively, 22% and 21%. These models additionally proved to be statistically significant (p < 0.001). In the group of girls, the somatic parameters and body composition components with the statistical significance in this regression model for the number of laps were age and FM%. At the same time, it is noted that age was found to be the strongest predictor of CRF in this group ($R^2$ = 16%), while FM% explains only 6% of the CRF level. A similar model was proposed by Emadeldin et al. studying body composition and its relationship to CRF among preschool children^52^ but the authors came to different conclusions. Their results show that the strongest predictors of CRF in their model were FM% and BMI, and the coefficient of determination ranked at 65%. Body fat percentage was also the strongest predictor of $\hbox {VO}_{2}$max in the study by Heileson et al.^53^ among people over 35 years of age. The linear regression model showed that for every 1% increase in FM%, VO_2_max decreased by 0,748 ml/kg/min. San Diego researchers came to identical conclusions. Body fat percentage proved to be the strongest predictor of CRF and aerobic capacity among forty active-duty soldiers^54^. Similar research results were also obtained by Forsse et al.^55^ who noted that as lean body mass grew, CRF increased, while decreasing FM and BF% resulted in higher CRF levels among people with chronic kidney disease. The studies by Ortega et al.^1^ also confirm that among individuals in whom high body weight is due to high obesity, $\hbox {VO}_{2}$max values are significantly lower in relation to body weight. It was also noted that as BMI increases, $\hbox {VO}_{2}$max decreases^1,42^. A different view was expressed by Bandyopadhyay and Chatterjee^56^, according to which adipose tissue can help increase its ability to capture $\hbox {O}_{2}$ for its aerobic metabolism. Adipose tissue begins to break down during exhaustive work to obtain energy through oxidative metabolism. Therefore, the $\hbox {O}_{2}$ demand is met by increased $\hbox {O}_{2}$ uptake, which is due solely to fat and not for FFM.

In the regression model which was proposed for the group of boys, age, BM, WHR and WHtR were taken into account. The coefficient of determination for these study group ranked at 21%. In this model the strongest predictor of CRF was WHtR ($R^2$ = 7%). However, coefficients of determinations of the remaining variables at a similar level, 6% for BM and 5% for WHR, respectively. It turns out that body shape assessed by WHtR is a crucial factor to affect CRF^51^. The regression models proposed by Zadarko-Domaradzka et al. showed that WHtR ($R^2$ = 50.0%) and WHR ($R^2$ = 39.2%) next to WC ($R^2$ = 47.1%), body fat (BF%, $R^2$ = 50.3%) and BMI ($R^2$ = 45.8%), were equally significant predictors of CRF^19^. In our research, indicators such as WC, HC or BMI were not significant predictors of CRF in any of the proposed models, except for the multiple regression model including all independent variables in the group of boys. A study conducted on a group of Iranian and Italian students shows a statistically significant relationship between WHtR and WHR and $\hbox {VO}_{2}$max^57^. Among Nigerian students, it was also noted that 60.2% of CRF was predicted by WHR^20^. There are increasing reports that WHtR is a much better predictor of CVD than BMI and WC^58,59^. At the same time, WHtR may be a more effective indicator of “early health risk” associated with central obesity^60^.

Summarizing our considerations, we can conclude that cardiorespiratory fitness should be an important element at the stage of school age, as high CRF can prevent abdominal and general obesity, as well as may be associated with maintaining health parameters in later life^61^. There is growing evidence that physical fitness, especially CRF, is linked to obesity and can counteract the negative consequences of childhood obesity^62,63^. These findings support the view that promoting high levels of cardiorespiratory fitness should begin in childhood. For this reason, schools and sports clubs should play an important role in promoting healthy lifestyles from an early age. Of course, this assessment is not a substitute for professional diagnosis, but it can serve as part of screening to identify the risk of STDs.

The strength of the article is undoubtedly the large group of adolescents studied. Our analysis of the literature showed that studies conducted on a population of Polish adolescents studied. very rarely focus on the relationship between CRF and body composition^19,64^. The main limitation of the study is the evaluation of CRF by an indirect method such as the 20m SRT. Although the test is widely used in many studies, direct methods are more accurate for assessing CRF. To verify the results more precisely, it would also be necessary to analyze the relationship between CRF and the level of physical activity of adolescents and to take into account the pubertal status of adolescents. Despite its limitations, the value of our study was in providing important data on the health of school-age children.

## Conclusions

It was shown that there are statistically significant relationships between cardiorespiratory fitness and selected somatic parameters and body composition components. In total sample CRF correlates most strongly with body fat content. This phenomenon is also observed according to the gender of the subjects. Therefore, it is worthwhile to focus on the assessment of this parameter, but not only with the help of BMI, which does not fully reflect body fat, but to use more precise methods, such as BIA. However, in the absence of the possibility of using specialized equipment, WHR and WHtR are helpful in estimating body fat distribution, the estimation of which does not require sophisticated equipment, and as it turns out, are important predictors of CRF.

## Practical applications

Identifying factors influencing the level of cardiorespiratory fitness can be used for early detection of current and future cardiovascular risk among adolescents. These research will allow for better health monitoring early in life. Additionally, it appears that high levels of cardiorespiratory endurance may play a key role in preventing general and abdominal obesity, therefore cardiorespiratory endurance should be an important target at a young age to prevent obesity and obesity-related health risks. Early identification of children with low levels of cardiorespiratory endurance is important and consideration should be given to adding assessment of this capacity to school health screenings. Primary care physicians, health care workers, teachers, and parents are potential recipients interested in implementing the results of this research.

## Data Availability

The datasets generated and/or analysed during the current study are available from the corresponding author upon reasonable request.
